# Waking up for defense! Melatonin as a regulator of stomatal immunity in plants

**DOI:** 10.1093/plphys/kiab481

**Published:** 2022-01-20

**Authors:** Javier Edgardo Moreno, Marcelo Lattarulo Campos

**Affiliations:** 1 Instituto de Agrobiotecnología del Litoral (UNL-Conicet), Facultad de Bioquímica y Ciencias Biológicas, Universidad Nacional del Litoral, Santa Fe, Argentina; 2 Integrative Plant Research Laboratory, Departamento de Botânica e Ecologia, Instituto de Biociências, Universidade Federal de Mato Grosso, Cuiabá, MT, Brazil

Melatonin is a tryptophan-derived compound discovered in the late 1950s as a molecule produced by the pineal gland of most vertebrates. Melatonin gained notoriety in the 1970s when it was demonstrated that its production increased in humans during the night in a rhythmic fashion associated with sleep synchronization (Xie et al., 2017). Melatonin is a ubiquitous molecule produced by bacteria, fungi, plants, and animals. In plants, melatonin regulates a wide range of processes, such as seed germination, root and shoot growth, and production of secondary metabolites, and is involved in circadian cycle regulation (Arnao and Hernández-Ruiz, 2019). The recent identification of a melatonin receptor named PHYTOMELATONIN RECEPTOR1 (AtPMTR1) in the plant model Arabidopsis (*Arabidopsis thaliana*) demonstrated that melatonin also governs an AtPMTR1-dependent mechanism of stomatal closure (Wei et al., 2018). As stomatal closure is a well-known process utilized by plants to restrain the invasion of pathogens, usually referred to as stomatal immunity (Melotto et al., 2006), melatonin is now gaining attention for its capacity to mediate responses to biotic stress in plants (Moustafa-Farag et al., 2020). Unfortunately, the underlying molecular mechanisms of melatonin regulation of plant immunity remain largely unknown.

In this issue of *Plant Physiology*, Yang et al. (2021) uncover aspects in the signaling pathway associated with melatonin-induced stomatal immunity in Arabidopsis and in the Chinese medicinal plant *Panax notoginseng*. The authors treated leaves of *P. notoginseng* with melatonin, followed immediately or 2 h later by inoculation with bacterial pathogen *Pseudomonas syringae* pv. *tomato* (*Pst*) DC3000. In both cases, melatonin treatment significantly reduced the bacterial growth in *P. notoginseng* leaves compared to control plants (mock treatment), indicating that melatonin modulates immunity. This result was partially explained by melatonin-induced stomatal closure observed in *P. notoginseng* leaves by 2 h after treatment, thus limiting the capacity of *Pst* to invade and infect the plant. As in a chemical arms race, *Pst* can produce coronatine, a phytotoxin that reverts the melatonin-induced stomatal closure, reopening these pores by 4 h after bacterial inoculation. Indeed, co-treatment of melatonin and coronatine inhibits the melatonin-induced stomatal closure in *P. notoginseng* leaves.

One of the key processes associated with stomatal immunity involves the activity of NADPH oxidases, enzymes involved in the production of reactive oxygen species (ROS) that function as secondary messengers to induce stomatal closure (Kwak et al., 2003; Li et al., 2020). To evaluate whether NADPH oxidases and ROS are involved in the melatonin-induced stomatal closure in *P. notoginseng*, the authors treated leaves with melatonin and catalase (a ROS scavenger) or melatonin and diphenyleneiodonium chloride (an inhibitor of NADPH oxidase). Both treatments decreased the stomatal aperture when compared to control plants treated only with melatonin, indicating that NADPH oxidase activity and ROS production are involved in stomatal closure induced by melatonin.

To further dissect the molecular machinery involved with this process, the authors used immunoblot experiments to identify two MAPK kinases, MPK3 and MPK6, whose melatonin-induced activation promotes stomatal closure in *P. notoginseng*. These results were confirmed using MPK3- and MPK6*-*inducible knockout mutants in Arabidopsis, where inactivation of the two MAPKs hinders the capacity of melatonin to induce stomatal closure and to mediate bacterial resistance. Previous studies demonstrated that melatonin binding to the AtPMTR1 receptor is required for melatonin-mediated stomatal closure in Arabidopsis (Li et al., 2020). Using an Arabidopsis *pmtr1* knockout mutant, the authors demonstrated that a functional melatonin receptor is necessary to activate MPK3 and MPK6 and consequently to promote stomatal closure upon melatonin perception. The role of PMTR1 in stomatal immunity was confirmed by pathogen infection assays using *Pst*, where the receptor knockout showed reduced bacterial resistance and reduced stomatal closure when compared to melatonin-treated control plants. By contrast, overexpression of PMTR1 in Arabidopsis led to enhanced bacterial resistance, even in the absence of melatonin. Their experiments with PMTR1 also led to the identification of an MPK3- and MPK6-independent molecular component that is necessary for melatonin-mediated stomatal immunity, the G protein α-subunit (GPA1). GPA1 is an important component of numerous signal transduction pathways, and it has been associated with stomatal closure before (Zhang et al., 2008). However, work by Yang et al. (2021) allowed evaluating how GPA1 participates in a more intricate signaling pathway that is activated upon melatonin treatment to induce stomatal immunity in plants (Figure  1).

**Figure 1:**
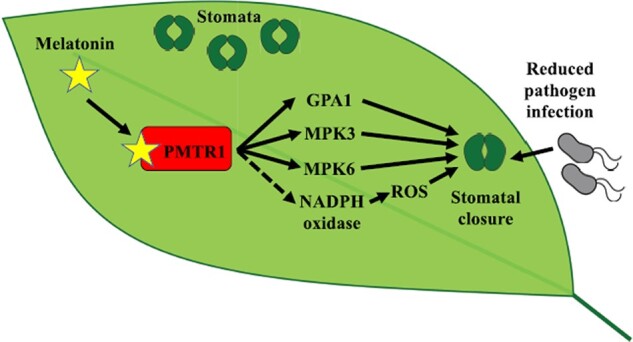
Melatonin as a regulator of stomatal immunity in plants. In this issue of *Plant Physiology*, Yang et al. (2021) dissect the molecular machinery involved with melatonin-induced stomatal closure in Arabidopsis and *P. notoginseng*. The authors demonstrate that melatonin binds to the melatonin receptor PMTR1 to activate a signaling cascade that includes GPA1 and activation of MAPKs MPK3 and MPK6 to induce stomatal closure and increase resistance to bacterial pathogen infection. They also demonstrate that melatonin-mediated stomatal closure depends on the activity of NADPH oxidase and ROS. The dashed line indicates a PMTR1-NADPH oxidase connection previously identified by the same research group (Wei et al., 2018).

Melatonin is currently one of the most popular medicines to treat sleep disorders in humans, but its effects on plant physiology are just now being uncovered. Work by Yang et al. (2021) provides a comprehensive overview on the signaling cascade activated by melatonin to induce stomatal immunity in *P. notoginseng* and Arabidopsis (Figure  1). Although additional components of the melatonin signaling pathway remain to be identified and more studies are necessary to understand how this molecular machinery is conserved in other plant species, this work already offers powerful insights for reanalyzing previous studies relating to the circadian clock and the induction of the defense response against pests and pathogens at different times of the day (Wang et al., 2011; Goodspeed et al., 2012). The potential role of melatonin keeping plants “awake” to biotic attack might be another path to enhance plant resilience in the near future.

*Conflict of interest statement.* None declared.

## References

[kiab481-B1] Arnao MB , Hernández-Ruiz J (2019). Melatonin: a new plant hormone and/or a plant master regulator? Trends Plant Sci 24: 38–48 30446305 10.1016/j.tplants.2018.10.010

[kiab481-B2] Goodspeed D , Chehab EW, Min-Venditti A, Braam J, Covington MF (2012) Arabidopsis synchronizes jasmonate-mediated defense with insect circadian behavior. Proc Natl Acad Sci USA 109: 4674–4677 22331878 10.1073/pnas.1116368109 PMC3311395

[kiab481-B3] Kwak JM , Mori IC, Pei ZM, Leonhardt N, Torres MA, Dangl JL, Bloom RE, Bodde S, Jones JDG, Schroeder JI (2003) NADPH oxidase *AtrbohD* and *AtrhbohF* genes function in ROS-dependent ABA signaling in *Arabidopsis*. EMBO J 22: 2623–2633 12773379 10.1093/emboj/cdg277 PMC156772

[kiab481-B4] Li D , Wei J, Peng Z, Ma W, Yang Q, Song Z, Sun W, Yang W, Yuan L, Xu X, et al (2020) Daily rhythms of phytomelatonin signaling modulate diurnal stomatal closure via regulating reactive oxygen species dynamics in *Arabidopsis*. J Pineal Res 65: e12640 10.1111/jpi.12640 32064655

[kiab481-B5] Melotto M , Underwood W, Koczan J, Nomura K, He SY (2006). Plant stomata function in innate immunity against bacterial invasion. Cell 126: 969–980 16959575 10.1016/j.cell.2006.06.054

[kiab481-B6] Moustafa-Farag M , Almoneafy A, Mahmoud A, Elkelish A, Arnao MB, Li L, Ai S (2020). Melatonin and its protective role against biotic stress impacts in plants. Biomolecules 10: 54 10.3390/biom10010054 PMC7022677 31905696

[kiab481-B7] Xie Z , Chen F, Li WA, Geng Z, Li C, Meng X, Feng Y, Liu W, Yu F (2017) A review of sleep disorders and melatonin. Neurol Res 39: 559–565 28460563 10.1080/01616412.2017.1315864

[kiab481-B8] Yang Q , Peng Z, Ma W, Zhang S, Hou S, Wei J, Dong S, Yu X, Song Y, Gao W, et al (2021) Melatonin functions in priming of stomatal immunity in *Panax notoginseng* and *Arabidopsis thaliana*. Plant Physiol 187: 2837–2851 34618091 10.1093/plphys/kiab419 PMC8644721

[kiab481-B9] Wang W , Barnaby JY, Tada Y, Li H, Tor M, Caldelari D, Lee DU, Fu XD, Dong X (2011) Timing of plant immune responses by a central circadian regulator. Nature 470: 110–114 21293378 10.1038/nature09766 PMC6601609

[kiab481-B10] Wei J , Li DX, Zhang JR, Shan C, Rengel Z, Song ZB, Chen Q (2018) Phytomelatonin receptor PMTR1-mediated signaling regulates stomatal closure in *Arabidopsis thaliana*. J Pineal Res 65: e12500 29702752 10.1111/jpi.12500

[kiab481-B11] Zhang W , He SY, Assmann SM (2008) The plant innate immunity response in stomatal guard cells invokes G-protein-dependent ion channel regulation. Plant J 56: 984–996 18702674 10.1111/j.1365-313X.2008.03657.x PMC2804871

